# Quantitative analysis of defect states in InGaZnO within 2 eV below the conduction band via photo-induced current transient spectroscopy

**DOI:** 10.1038/s41598-023-40162-0

**Published:** 2023-08-17

**Authors:** Hyunmin Hong, Min Jung Kim, Dong-Joon Yi, Yeon-Keon Moon, Kyoung-Seok Son, Jun Hyung Lim, KwangSik Jeong, Kwun-Bum Chung

**Affiliations:** 1 Division of Physics and Semiconductor Science, Dongguk University, Seoul, 04620 Republic of Korea; 2 Department of Display R&D Center, Samsung Display, Yongin, 17113 Republic of Korea

**Keywords:** Materials science, Physics

## Abstract

This work investigates the function of the oxygen partial pressure in photo-induced current measurement of extended defect properties related to the distribution and quantity of defect states in electronic structures. The Fermi level was adjusted by applying a negative gate bias in the TFT structure, and the measurable range of activation energy was extended to < 2.0 eV. Calculations based on density functional theory are used to investigate the changes in defect characteristics and the role of defects at shallow and deep levels as a function of oxygen partial pressure. Device characteristics, such as mobility and threshold voltage shift under a negative gate bias, showed a linear correlation with the ratio of shallow level to deep level defect density. Shallow level and deep level defects are organically related, and both defects must be considered when understanding device characteristics.

Due to the continuous growth of the Internet of Things (IoT) technology, various transistors, solar cells, light-emitting diodes, and sensors have been miniaturized and integrated^1^. Accordingly, the production processes are diversified, the structure of the device is complicated, and the defects are increasing. Among various materials used in devices, an amorphous oxide semiconductor (AOS) is an essential compound of a semiconductor, because of superior electrical properties, low-temperature fabrication process, and high optical transparency compared to conventional silicon-based TFTs^2,3^. Hence, vacancies, inserts and substitutes can act as defective elements^4,5^. These oxide semiconductor defects can act differently as donors or trap sites depending on the energy level^6^. Defects that act as electron traps create local barriers, increase carrier scattering, interfere with drift currents and induce diffusion currents^7^. Therefore, it is significant to quantitatively measure the defect density and activation energy in order to analyze the device characteristics.

Defects in AOS thin films can exist at several energy levels and may have more defects than previously reported^8,9^. However, the measurable range of the defect density and activation energy is limited due to differences in electrical and optical reactivity according to materials^10^. In addition, there is no suitable methodology that can directly analyze the interfacial state defects between each structural layer, which is determined to exist in the device. For example, with the charge pump method can measure the defect density and activation energy through modeling by applying a gate voltage as a pulse to device. However, since the band bending as a function of gate voltage varies depending on the device structure and channel properties, the measurement resolution also varies. Deep level transient spectroscopy (DLTS) can also quantitatively measure defects by analyzing the change in capacitance during charge/discharge as a function of temperature^11,12^. In this method, direct comparison with device characteristics of the TFT structure is difficult because the electrode must be made a vertical Schottky contact to measure the accurate capacitance. In our last study, we were able to quantitatively measure defects only a few hundred meV away from the conduction band minimum^13^. Therefore, there is a need for a method that can quantitatively measure defects distributed at various levels in the bandgap.

In this paper, the Fermi level of a-IGZO TFTs is adjusted by applying a negative gate bias to extend the range of quantitatively analyzed using photo-induced current transient spectroscopy (PICTS). An improved machine learning technique with high resolution, fast analysis, and reliability was used for the analysis of the large amounts of data obtained by measurement. In addition, the physical origin of defect states was investigated through density functional theory calculations. The characteristics of the device were analyzed through the measured defect, and the role of the defect was investigated.

## Experiment

In the fabrication of the bottom gate structure of a-IGZO TFTs, a heavily doped *p*-type Si wafer and 100-nm-thick SiO_2_ layer served as the respective gate electrode and gate insulator. A 20 nm-thick a-IGZO active layer was deposited by radio frequency (RF) magnetron sputtering from an IGZO (In:Ga:Zn = 1:1:1 at. %) target at room temperature (300 K). An important parameter during deposition is the oxygen partial pressure (${\mathrm{P}}_{{\mathrm{O}}_{2}}$). This indicates the flow rate of oxygen relative to the total flow rate of all gasses present. We fabricated three TFTs with different oxygen partial pressures to investigate how this would affect the characteristics of the resulting devices. During, the deposition of the a-IGZO layer, the ${\mathrm{P}}_{{\mathrm{O}}_{2}}$ was set to 0, 10, or 60% in the sputtering process. Then, 100 nm-thick ITO was patterned with a metal shadow mask as the source/drain (width/length = 800/200 um) by DC magnetron sputtering. Finally, the a-IGZO TFTs were annealed at 350 ℃ for 1 h in an ambient air atmosphere using a furnace system.

**Figure 1:**
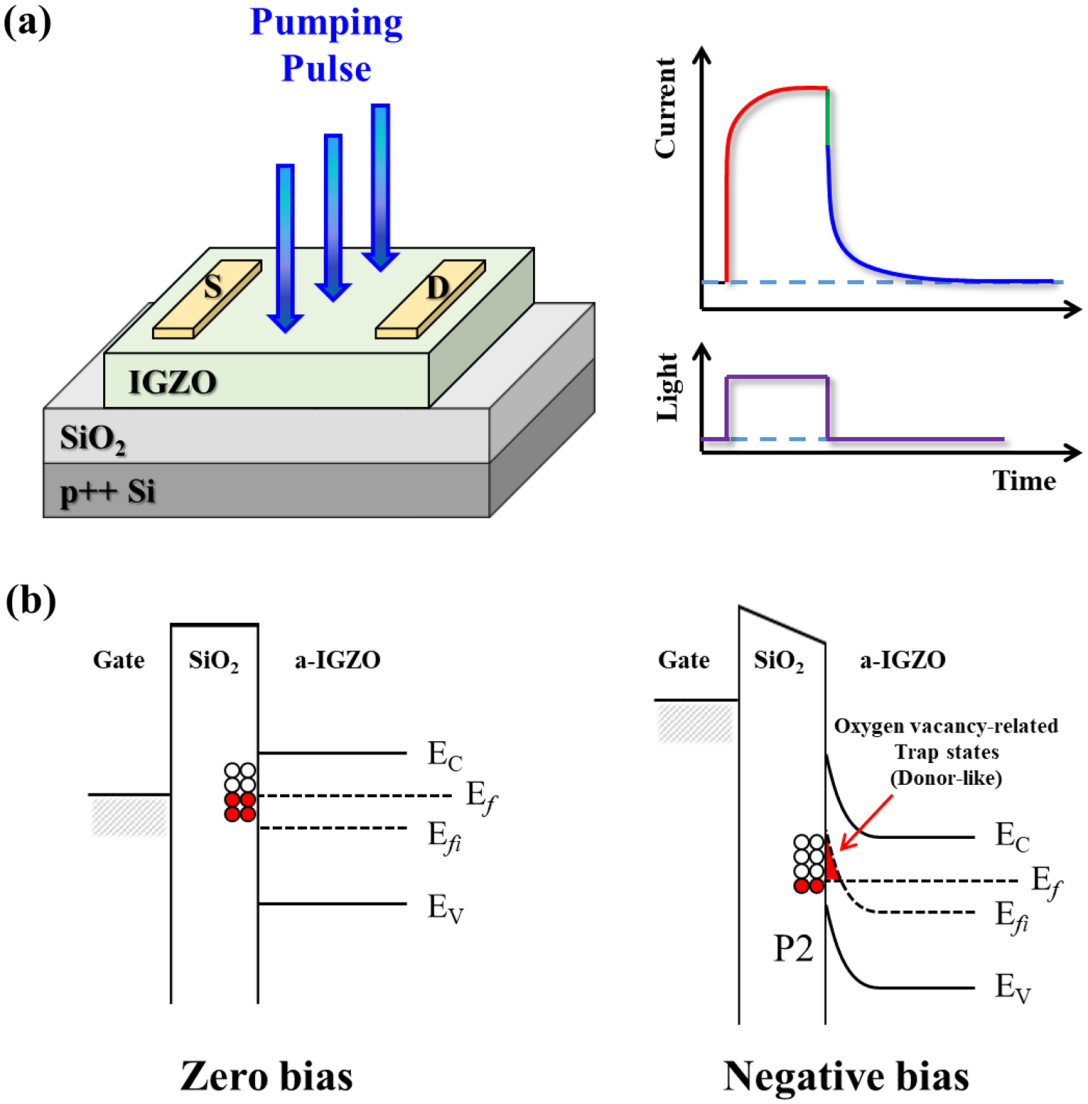
(**a**) PICTS experimental principle schematic diagram, (**b**) band bending of the a-IGZO TFT under gate bias.

Figure 1a shows a schematic diagram of the experimental set-up. To quantitatively analyze the defect states using PICTS, UV-LEDs (λ = 275 ± 12 nm) periodically irradiated the a-IGZO TFTs using a pulse generator. In order to measure the photo responses according to the LED to occur only in the channel, the size of the light was adjusted to a circular shape with a diameter of 100 µm through × 10 magnification microscope and focused on the channel region. When the LED pulse is irradiation on the channel of the device, electrons in the valence band are excited to the conduction band and cause a local increase in conductivity dependent on the generation and recombination processes. After the LED pulse is turned off, the signal is rapidly reduced due to the rapid recombination of excess carriers generated. This is followed by a slower decaying part because carriers trapped in the defect state are re-emitted^14^. Analyzing this signal with the Laplace transform can directly extract the decay constant of the detrapped carriers. The detrap of these carriers is determined by the concentration and energy trapped in the defect and is dependent on the measurement temperature^15^. Therefore, if the time constant is analyzed after measuring the transient current at various temperatures, the concentration and activation energy of the defect can be calculated. In this paper, the temperature was controlled in the range of 77 to 300 K with liquid nitrogen. Figure 1b shows the band bending corresponding to the negative gate bias. When zero gate bias is applied to the gate of the a-IGZO TFT, the Fermi level (E_F_) of a-IGZO and gate are balanced, and some defect states at the top of E_F_ are unoccupied. When a negative bias is applied, the E_F_ is shifted toward the center of the bandgap due to band bending, and most defect states are unoccupied^16^. PICTS is a method to measure the transient current when the electrons trapped in the defect are released after the unoccupied defect state is filled with light^17^. Therefore, when a negative gate bias is used to clear the occupied defect state by changing the E_F_, the PICTS can be used to measure the defects at an extended level.

The device characteristics of the a-IGZO TFTs were measured using a semiconductor-parameter analyzer. Drain current (I_D_) was measured under a gate voltage (V_G_) sweep from −10 to 90 V.

## Calculation method

To predict the generation and role of oxygen-related defects in the IGZO system, we performed density functional theory (DFT) calculations with the Vienna ab-initio simulation package (VASP) and the PBEsol functional^18,19^. First, we generated three amorphous models by a melt-quench process with an ab-initio molecular dynamics (MD) simulation. During the MD simulation, an NPT ensemble with a Langevin thermostat, gamma K-point, and 500 eV cut-off energy is used^20^. One model contains 20 Indium, 20 Gallium, 20 Zinc, and 80 oxygen atoms to represent IGZO in 1:1:1:4 stoichiometry, while other models contain 20 Indium, 20 Gallium, 20 Zinc, and 79 or 78 oxygen atoms to represent oxygen deficiency IGZO. In the first stage, both systems are simulated to melt in 3000 K for 12 ps with a 3 fs step. After melting, the MD simulation is run to cool from 2000 to 300 K in 50 K/ps during 54 ps with a 3 fs step. During MD simulation, gamma K-Point and 500 eV cut-off energy are used. After the MD simulation, systems are geometrically optimized until the 0.01 eV/Å condition is satisfied. In the geometric optimization 2 $\times$ 2 $\times$ 2 k-points ,500 eV cut-off energy and PBESol functional are used. From optimized structure, electronic structure of IGZO calculated with 2 $\times$ 2 $\times$ 2 k-points, 500 eV cut-off energy and HSE06 hybrid functional^21^. Formation energy of oxygen deficiency (${\mathrm{E}}_{\mathrm{oxygen}}$) in neutral IGZO is calculated with assumption that oxygen atoms are dissociated from IGZO and the formation of n oxygen molecule (O_2_).

$$ {\text{E}}_{{{\text{oxygen}}}} = {\text{ E}}\left( {{\text{In}}_{20} {\text{Ga}}_{20} {\text{Zn}}_{20} {\text{O}}_{{80 - {\text{n}}}} } \right){ } - {\text{ E}}\left( {{\text{In}}_{20} {\text{Ga}}_{20} {\text{Zn}}_{20} {\text{O}}_{80} } \right){ } + { }\frac{{\text{n}}}{2}{ } \times {\text{ E}}\left( {{\text{O}}_{2} } \right) $$

To simulate the charging effect on IGZO, calculations with -2, -1, 0, 1, 2 charging states were performed with same conditions. Formation energy of charging states (${\mathrm{E}}_{\mathrm{charging}}$) to reference with neutral states are calculated from following equation^22^ 

$$ {\text{E}}_{{{\text{charging}}}} = {\text{E}}\left( {\text{q}} \right) - {\text{E}}\left( {\text{n}} \right) + {\text{q}}\left( {\upmu _{{\text{e}}} + \Delta {\text{V}}} \right) $$

where E(q) is the total energy of the supercell with charge q, E(n) is the total energy of a neutral supercell, $\upmu$_e_ is the electron chemical potential, and $\Delta$V is the energy level shift of the valence band maximum. Therefore, the total formation energy versus the electron chemical potential is calculated from the equation.

$$ {\text{E}}_{{{\text{total}}}} = {\text{E}}_{{{\text{oxygen}}}} + {\text{E}}_{{{\text{charging}}}} $$

## Results and discussion

**Figure 2:**
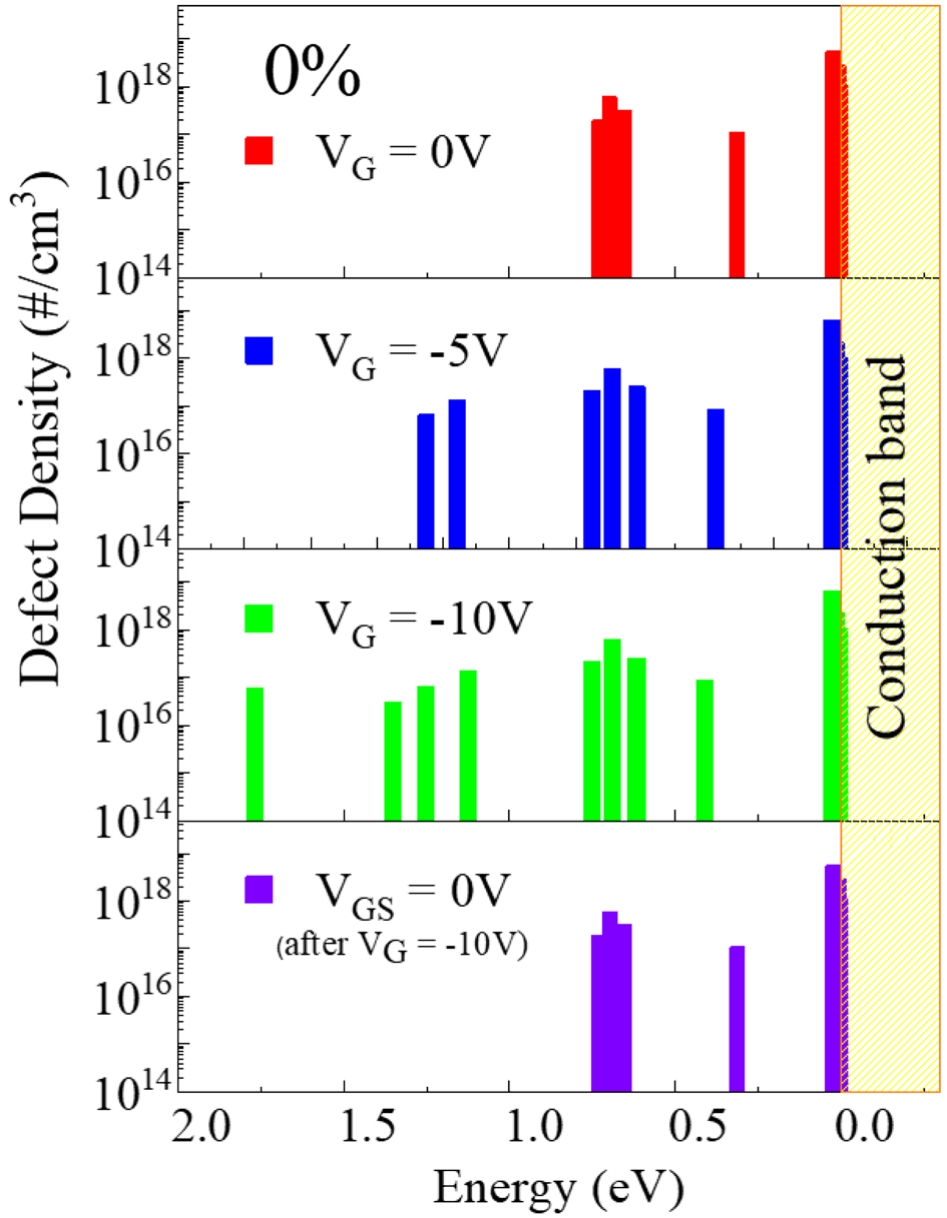
Changes in the activation energy and defect density before and after applying the negative gate bias of an a-IGZO TFT with an oxygen partial pressure of 0%.

Figure 2 shows the quantitatively measured defect density and activation energy before and after applying a negative gate bias to a-IGZO TFT with ${\mathrm{P}}_{{\mathrm{O}}_{2}}$ of 0%. When a zero gate bias was applied, defects were measured to < 1.0 eV from the conduction band. Defects were additionally measured to < 1.5 eV from the conduction band after applying a negative gate bias of −5 V and further to < 2.0 eV from the conduction band after applying a negative gate bias of −10 V. As the gate bias changed from 0 to −5 to −10 V, the defects density did not change and as the gate bias increased, only the defect measurable range increased. After measuring again with a gate bias of zero, the activation energy and defect density were the same as the originally measured values. Thus, it can be seen that the measurable range expands due to the change in Fermi level when a negative gate bias voltage is applied without changing the physical/chemical properties of the channel.

**Figure 3:**
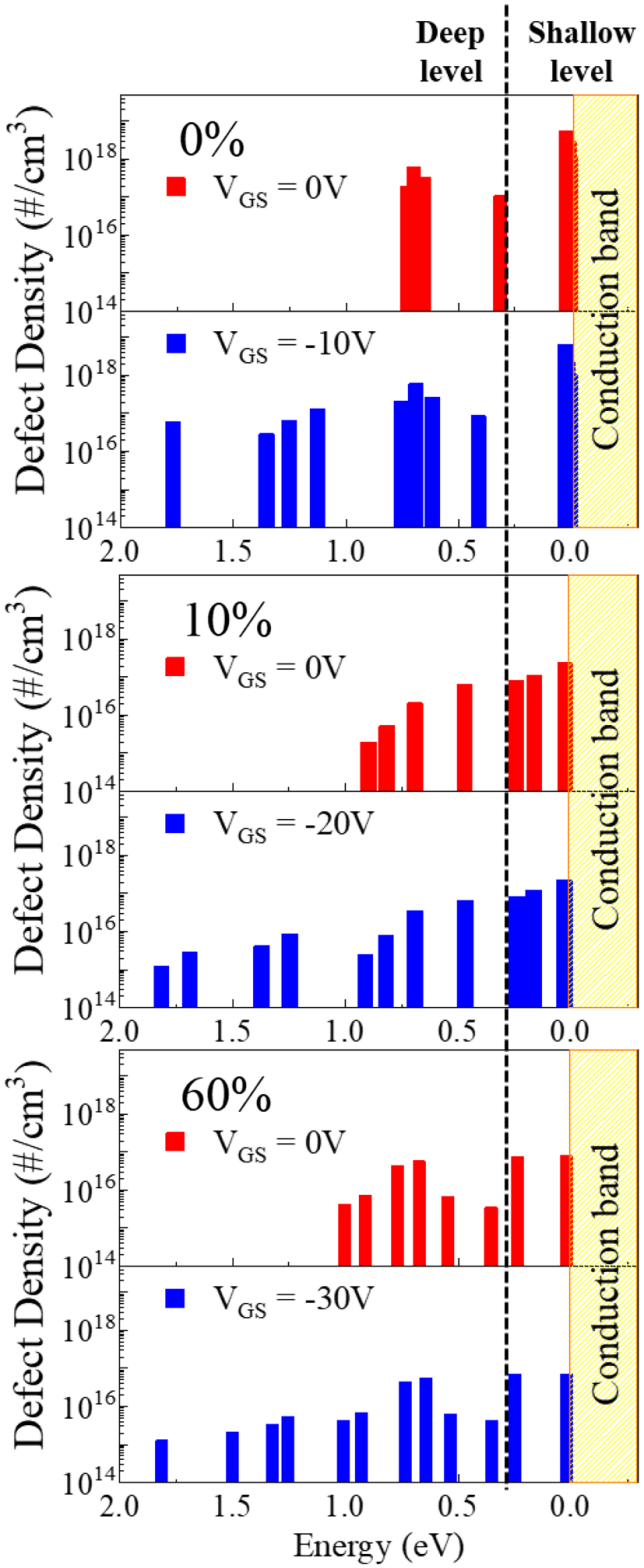
The extended defect measurement region by applying the negative gate bias of a-IGZO TFTs as a function of the oxygen partial pressure.

Figure 3 shows the extended defect measurement range when negative gate bias of a-IGZO TFTs is applied as a function of ${\mathrm{P}}_{{\mathrm{O}}_{2}}$ for the negative gate bias voltage applied to a-IGZO TFT, the maximum value at which device breakdown does not occur when UV light is applied was : −10 V, −20 V, and −30 V, respectively. In all a-IGZO TFTs, the defect measurement range extended to < 2.0 eV when negative gate bias voltage was applied. The defect density of the shallow and deep levels decreased as the ${\mathrm{P}}_{{\mathrm{O}}_{2}}$ increased. These changes in defects can affect the device characteristics. All state of defects within the bandgap can act as electron trapping and scattering sites and affect the mobility of the device, but they can have different roles and effects on the TFT depending on the defect density and activation energy. From previous studies, shallow defect level and deep defect level can be classified based on ~ 0.25 eV at the conduction band minimum^23,24^. When the activation energy is less than 0.25 eV in the conduction band, it is a shallow level defect, and the electrons trapped in this defect can be easily detrapped at room temperature and act as charge carriers. When the activation energy is above 0.25 eV in the conduction band, it is a deep level defect, in which electrons are trapped^25^. The trapped electrons are released during device operation and can increase the scattering of charge carriers and decrease their mobility.

**Figure 4:**
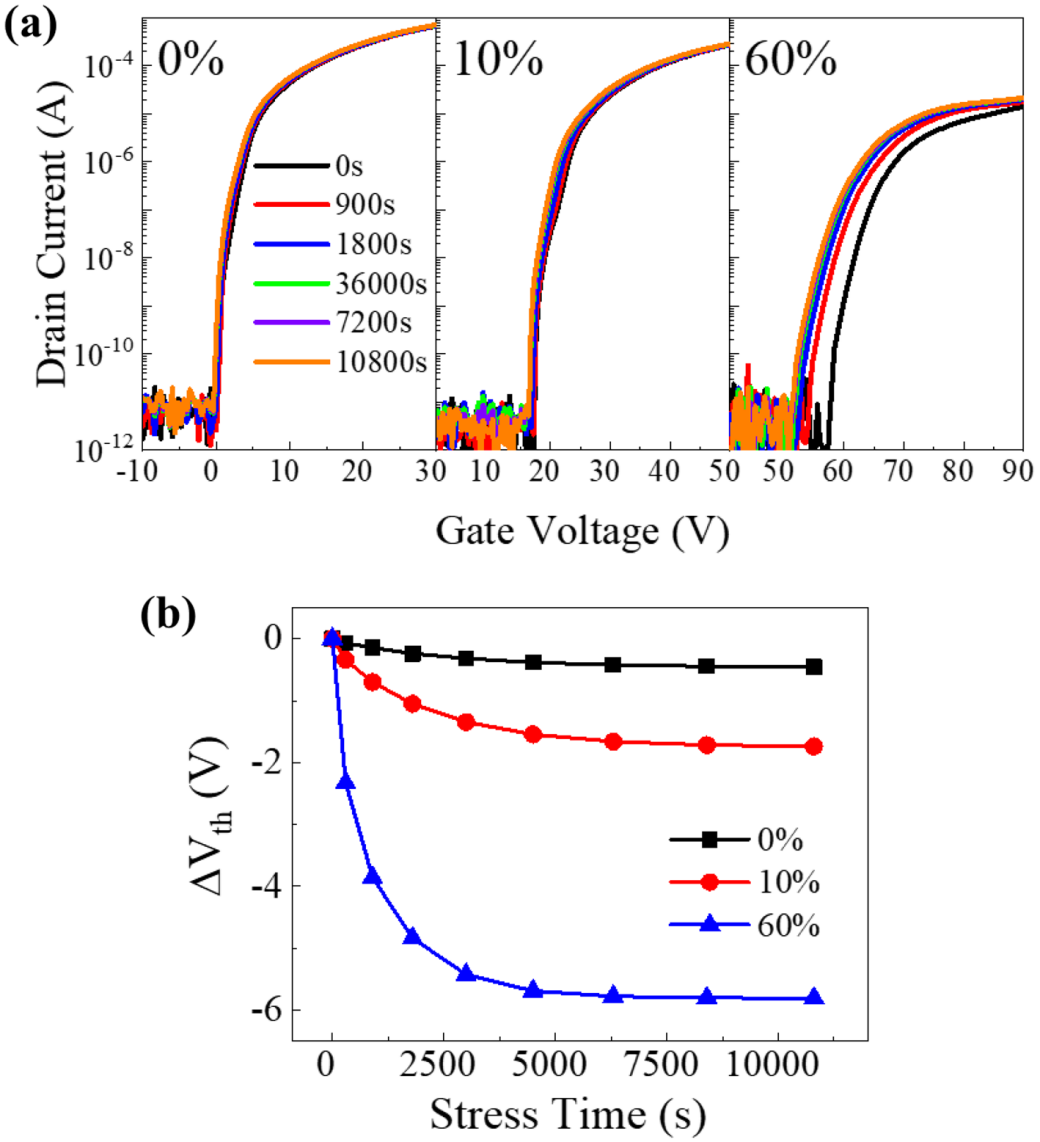
(**a**) Transfer characteristics, (**b**) shift of the a-IGZO TFTs threshold voltage under the negative gate bias stress as a function of the oxygen partial pressure.

Figure 4a and b show the shift in the transfer curve and the change in threshold voltage (V_th_) of the a-IGZO TFTs as a function of ${\mathrm{P}}_{{\mathrm{O}}_{2}}$ under a negative gate bias of −20 V for 10800 s. As ${\mathrm{P}}_{{\mathrm{O}}_{2}}$ increased from 0 to 10 to 60%, the threshold voltage (V_th_) increased from 2.02 to 20.07 to 57.67 V. These increases were due to a decrease in carrier concentration on the channel. The μ_FE_ decreased from 15.05 to 5.51 to 0.46 cm^2^ /V∙s, and the subthreshold swing (S.S.) increased from 0.44 to 0.83 to 1.52 V/decade as the ${\mathrm{P}}_{{\mathrm{O}}_{2}}$ increased from 0 to 10 to 60%, respectively. The TFTs fabricated with higher ${\mathrm{P}}_{{\mathrm{O}}_{2}}$ showed a larger shift in the negative direction of the transfer curve without any change in the μ_FE_ and S.S. values. Device performance degradation, such as increased S.S. value, decreased mobility, and increased bias instability (∆V_th_), is affected by defect density and its activation energy. From the results of previous studies, it is known that the supply of supplemental oxygen during the process reduces the defects in the channel and improves the device characteristics^26,27^. However, the total defect density decreased to 1.08 × 10^19^_,_ 5.41 × 10^17^, and 2.85 × 10^17^ cm^−3^ as the ${\mathrm{P}}_{{\mathrm{O}}_{2}}$ increased, but the device characteristics deteriorated. Therefore, to understand the TFT properties, a detailed analysis of the channel defects is required.

**Figure 5:**
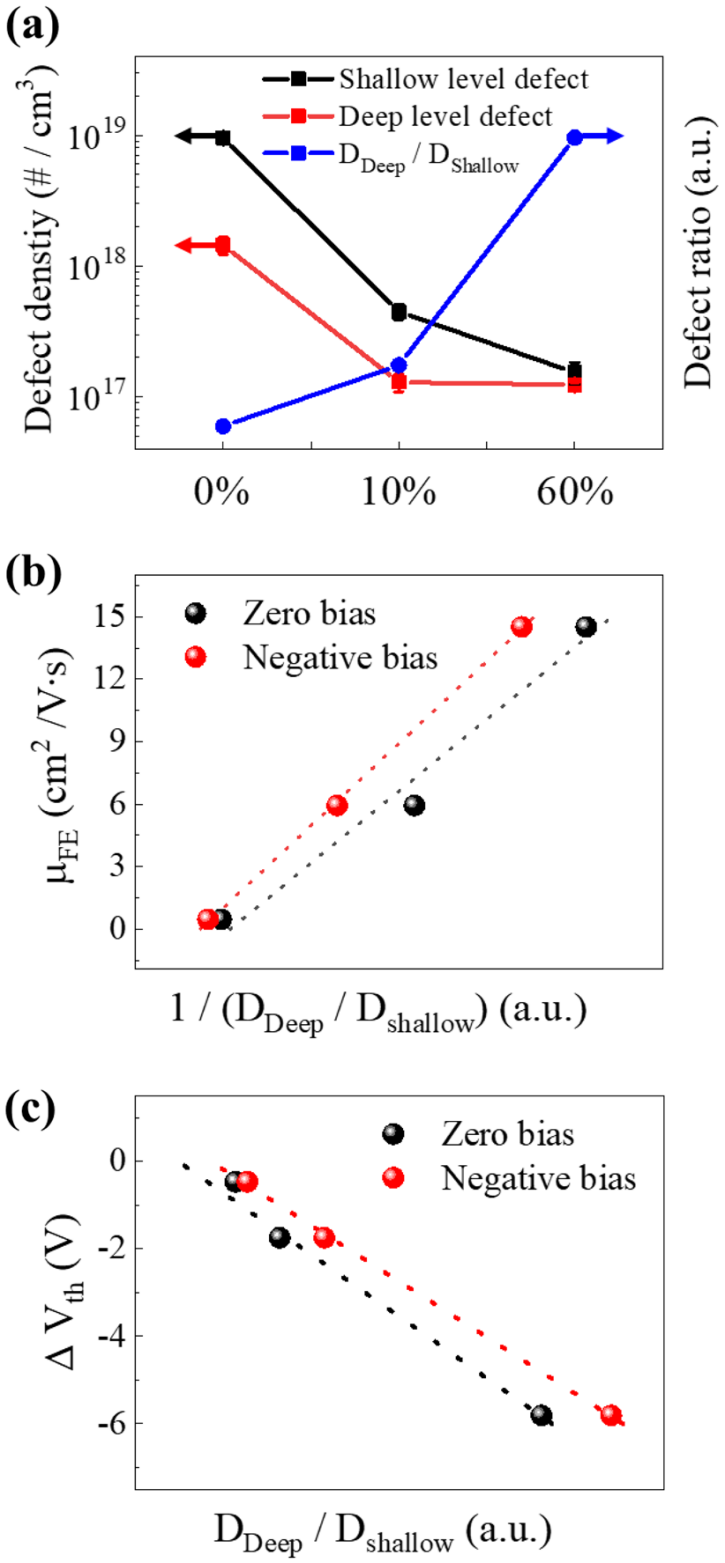
(**a**) Shallow level defect density, deep level defect density, and defect ratio, (**b**) correlation between defect ratio and field effect mobility, and (**c**) correlation between defect ratio and shift of threshold voltage under the negative gate bias stress of the a-IGZO TFTs as a function of the oxygen partial pressure.

Figure 5a shows the concentration of defects on the shallow and deep level defects and the ratio of each defect as a function of deposition oxygen partial pressure. As the ${\mathrm{P}}_{{\mathrm{O}}_{2}}$ increased from 0 to 10 to 60%, the density of shallow level and deep level defects decreased. In previous studies, the carrier concentration in the channel decreases as the shallow level of defects decreases. This coincided with the result that V_th_ increased in the characteristics of the device as the ${\mathrm{P}}_{{\mathrm{O}}_{2}}$ increased. However, as the ${\mathrm{P}}_{{\mathrm{O}}_{2}}$ increased, the mobility of the device decreased despite the decrease in deep-level defects. This shows a different trend from conventional interpretations of deep level defect and mobility correlations. Therefore, a new interpretation of the channel defect analysis is needed, which means that the defects are strongly interconnected with each other. It is necessary to compare device characteristics based on the relative density ratio (${D}_{Deep}/{\mathrm{D}}_{Shallow}$) of deep level defect region and shallow level defect region. Figure 5b and c show mobility as a function of ${D}_{Deep}/{D}_{Shallow}$ and ΔV_th_ characteristics in NBS. The ${D}_{Deep}/{D}_{Shallow}$ and mobility are in inversely related meaning that the greater the influence of the deep level defect compared to the carrier concentration or shallow level defect in the device, the lower the mobility. As the ${D}_{Deep}/{D}_{Shallow}$ increases, the reliability of the device deteriorates, which means that a deep level defect in the thin film will affect the reliability of the device. Moreover, when comparing the ${D}_{Deep}/{D}_{Shallow}$ and the device characteristics at zero bias voltage and negative bias voltage, a linear trend was clearly observed when the measurement range was extended by applying negative bias voltage to the device gate. Therefore, the characteristics of the TFT can be more accurately described by the extended defect measurement.

**Figure 6:**
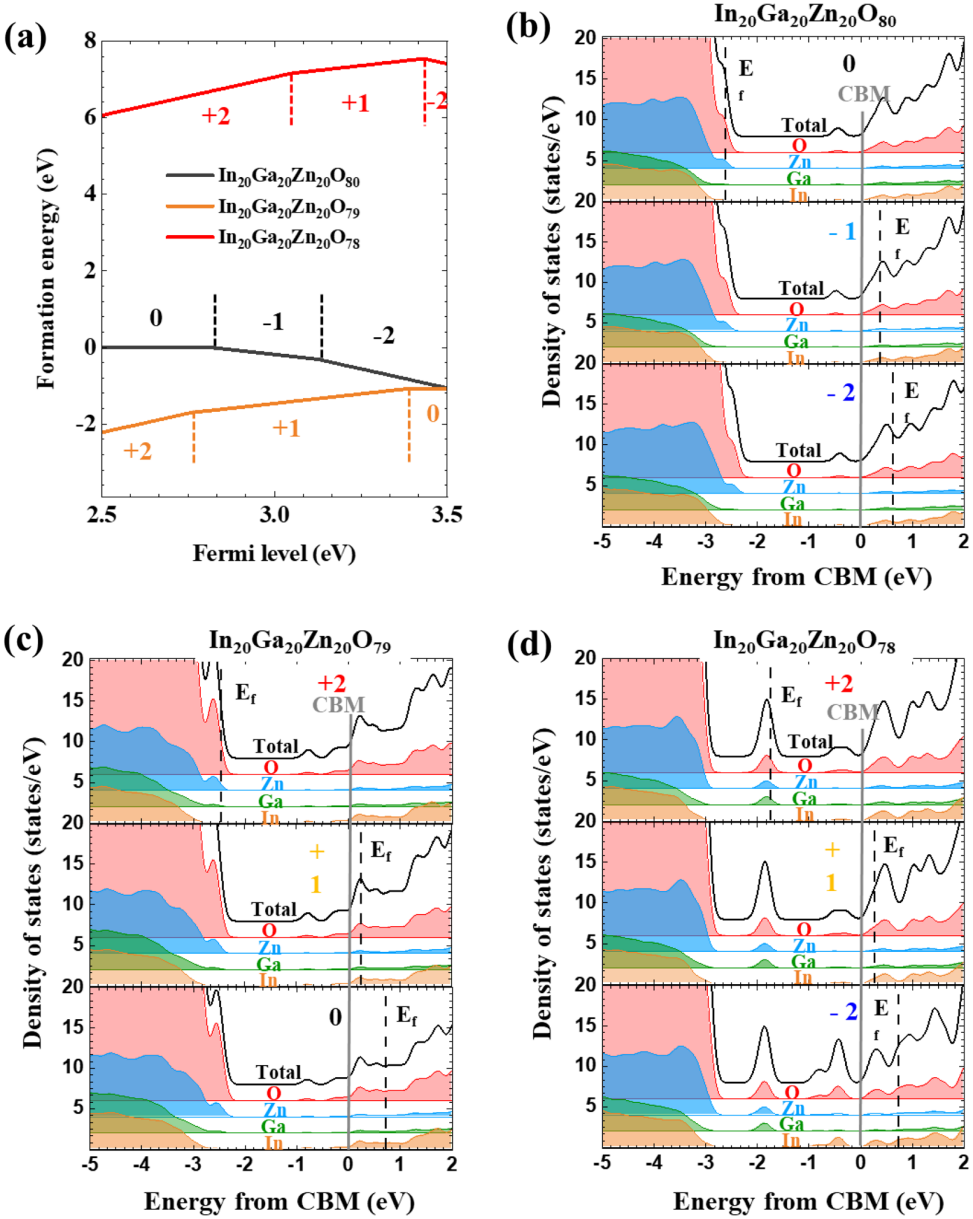
(**a**) Formation energy for in stoichiometry (In_20_Ga_20_Zn_20_O_80_) and oxygen deficient IGZO (In_20_Ga_20_Zn_20_O_79_ and In_20_Ga_20_Zn_20_O_78_). (**b**) Density of states for In_20_Ga_20_Zn_20_O_80_ (**c**) density of states for In_20_Ga_20_Zn_20_O_79_ (**d**) density of states for In_20_Ga_20_Zn_20_O_78_.

Figure 6a shows the formation energy of charged states as a function of the Fermi level (chemical potential of an electron). As shown in figure (a), when one (In_20_Ga_20_Zn_20_O_79_) or two (In_20_Ga_20_Zn_20_O_78_) oxygen atoms are deficient in the IGZO system, + 2 and + 1 states are stable. In the IGZO system, oxygen gap (oxygen deficiency) acts as a donor introducing electrons into the system and becoming positively charged. Considering the Fermi level of IGZO, + 1 charge states dominate in IGZO, when it is synthesized with ${\mathrm{P}}_{{\mathrm{O}}_{2}}$ 0%. Figure 6b–d show the change in the density of states from CBM according to the amount of oxygen in InGaZnO. When the Fermi level decreases by increasing the partial pressure of oxygen during deposition or gate bias, the fraction of + 2 charge states becomes larger^28^. By lowering the Fermi level in the system, deep level defect states which raise from oxygen deficiency become empty states and scattering sources for charge carrier transport. Therefore, when the oxygen partial pressure increases, the fraction of defects in the deep levels increases while the total number of defect states decreases. The position of the defect states at a deep level is 1.0 eV (one oxygen deficient) which is mainly originated from, and 1.8 eV (two oxygens deficient) away from the CBM, which is consisted with the experimental values. The extent of oxygen deficiency in the system is related to the oxygen partial pressure during synthesis. As can be seen from the results DFT, the oxygen-related defects can simultaneously modulate both the state fraction and the preferred charge states.

**Figure 7:**
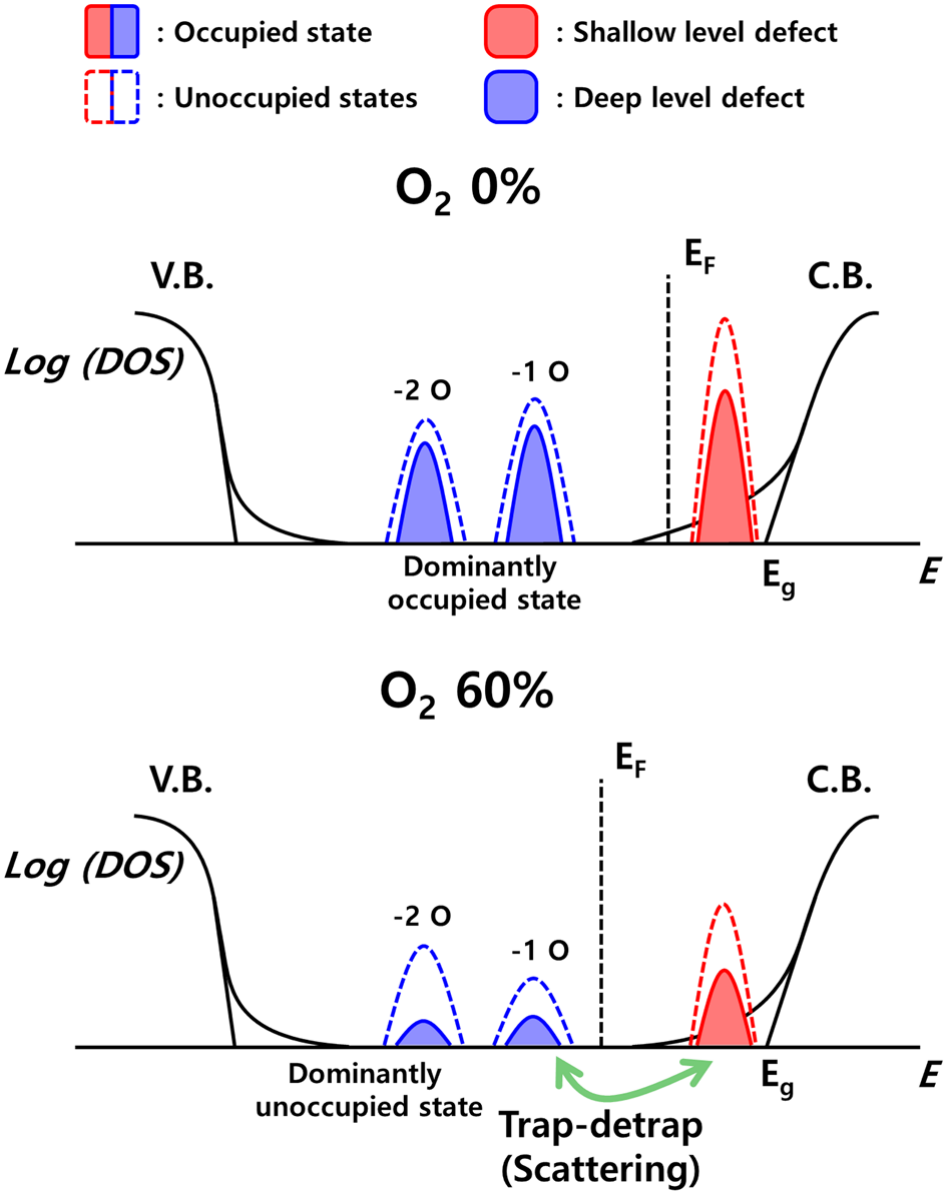
Schematic energy band diagram of the defect state in IGZO TFTs with different oxygen partial pressure.

Figure 7 shows a schematic energy band diagram of the defect state in IGZO TFTs with different ${\mathrm{P}}_{{\mathrm{O}}_{2}}$. As the ${\mathrm{P}}_{{\mathrm{O}}_{2}}$ increases, the total density of defect states and the Fermi level decrease, so that deep level defects may dominantly exist in an unoccupied state. Also, the ratio of IGZO with two oxygen deficient states increases, resulting in the ratio of states 2.0 eV away from the CBM increasing, while the ratio of states 1.0 eV away from the CBM decreases. Therefore, the ratio for the + 2 charge state increases, which traps more electrons and increases the scattering source. These results indicate that the mobility and reliability of the device deteriorate as the ${\mathrm{P}}_{{\mathrm{O}}_{2}}$ increased.

## Conclusion

In summary, the extended defect characteristics related to the distribution and amount of defect states in electronic structures as a function of oxygen partial pressure (${\mathrm{P}}_{{\mathrm{O}}_{2}}$ either 0%, 10%, or 60%) were investigated. By applying a negative gate bias, it was possible to extend the measurable range of activation energy to 2.0 eV. The device characteristics showed a linear correlation with the ratio of the shallow and deep levels density. Therefore, the ratio of the two defect states is closely related to the electrical properties and the properties of the device. By calculations through density functional theory, the origin and behavior of defect states in the region of activation energy of 2.0 eV are explained. Adjusting the amount of oxygen deficiency, simultaneously adjusting the density of defect states and the Fermi level, and modulating preferred charging states (ratio of empty states).

### Supplementary Information

Supplementary Information.

## Data Availability

The datasets that support the findings of this study are available from the corresponding author upon reasonable request.
