# Supplementary material for: Quantitative analysis of defect states in InGaZnO within 2 eV below the conduction band via photo-induced current transient spectroscopy
Source: Sci Rep. 2023 Aug 17;13:13407. doi: 10.1038/s41598-023-40162-0 (PMC10435584; doi:10.1038/s41598-023-40162-0)
Supplement: Supplementary file 1 — Supplementary Information. [file 41598_2023_40162_MOESM1_ESM.docx]

**Appendix**

Figure A 1 shows XPS spectra for the O 1*s* states of the a-IGZO thin-films as a function of $P_{O_{2}}$. For a detailed analysis of the chemical-bonding states, the O 1*s* spectra were deconvoluted with three Gaussian peaks. A strong peak at low binding energy (O1) at 531 eV is related to the oxygen ions on the metal oxide without an oxygen vacancy, which indicates In-O, Ga-O, and Zn-O bonds.^^[[1]](#endnote-1)^^ The medium binding energy peak (O2) at 532 eV is associated with an oxygen vacancy. Most of the time, chemically adsorbed or dissociated up metal-hydroxide is thought to be cause of the high binding energy peak (O3) at 533 eV.^^[[2]](#endnote-2)^^ The relative area of the O1 peak has increased, whereas the relative area of the O2 peak has decreased with increasing$P_{O_{2}}$. These changes could be associated with an increase in metal-oxygen bonds and a decrease in Vo during the deposition process, which corresponds to a decrease of the free carrier in the a-IGZO.^^[[3]](#endnote-3)^^

Figure A 2 shows the band edge states as a function of $P_{O_{2}}$. The band edge states below the conduction band were deconvoluted using Gaussian peaks. The Gaussian peaks consist of two distinct states, the defect states in the shallow level (D1) and those in the deep level (D2). All defects within the bandgap can act as electron trapping and scattering sites and affect the mobility of the device, but since D1 is near to the conduction band, it can be easily excited at room temperature. Therefore, electrons trapped in D1 can be detrapped in a shorter time than in D2, and it affects carrier concentration rather than mobility. As the $P_{O_{2}}$ increased from 0 to 10 and 60%, the area of D1 and D2 decreased. These changes are consistent with the quantitative results measured by PICTS.


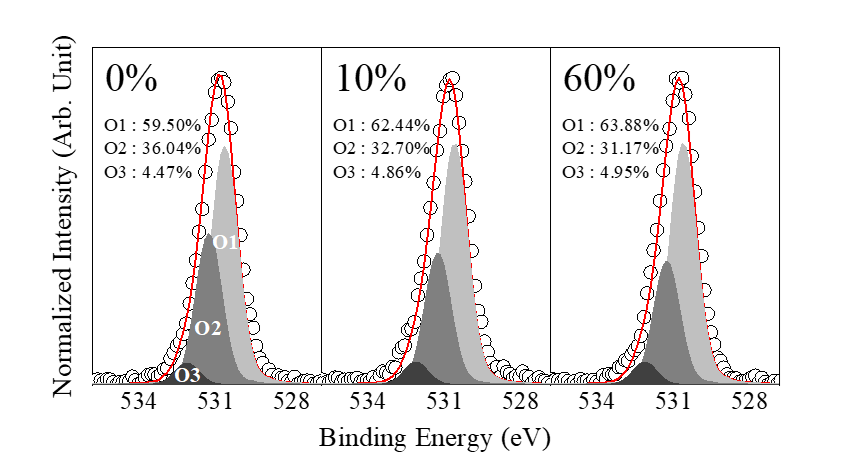


Figure A 1 XPS spectra of O 1s state of the a-IGZO thin-films as a function of the oxygen partial pressure


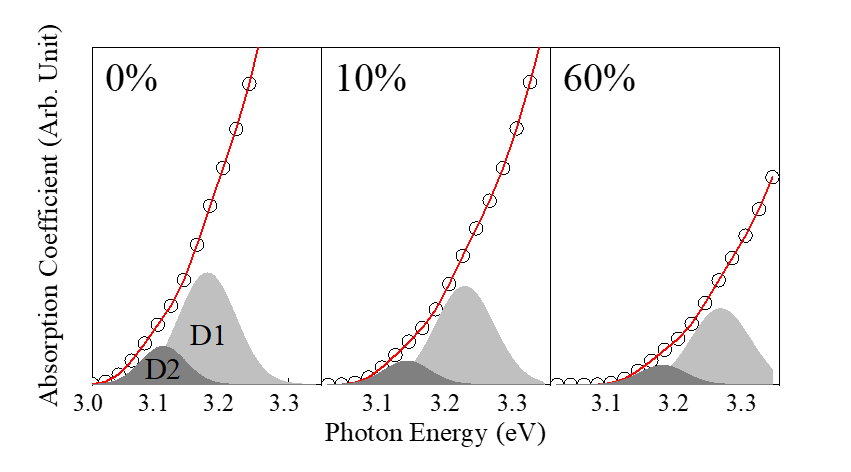


Figure A 2 The enlargement of band edge states below the conduction band. The two deconvoluted peaks represent the defect state in shallow level (D1) and deep level (D2).

## **References**

1. C. Donley, D. Dunphy, D. Paine, C. Carter, K. Nebesny, P. Lee, D. Alloway, N. R. Armstrong, Langmuir, 2002, **18**, 450 [↑](#endnote-ref-1)
2. B. D. Ahn, H. S. Shin, H. J. Kim, J. -S. Park, J. K. Jeong, Appl. Phys. Lett. 2008, **93**, 203506 [↑](#endnote-ref-2)
3. H. W. Park, M. J. Choi, Y. Jo, K. B. Chung, Appl. Sur. Sci. 2014, **321**, 520 [↑](#endnote-ref-3)
